# High levels of pretreatment CA125 are associated to improved survival in high grade serous ovarian carcinoma

**DOI:** 10.1186/s13048-016-0247-6

**Published:** 2016-07-07

**Authors:** Flavia Morales-Vásquez, Enrique Pedernera, Jesús Reynaga-Obregón, Horacio Noé López-Basave, María José Gómora, Elisa Carlón, Sandra Cárdenas, Raúl Silva-Ayala, Miguel Almaraz, Carmen Méndez

**Affiliations:** Medical Oncology Department, National Cancerology Institute, Mexico City, Mexico; Embryology Department, School of Medicine, National Autonomous University of Mexico (Universidad Nacional Autonoma de Mexico-UNAM-), Mexico City, Mexico; Public Health Department, School of Medicine, National Autonomous University of Mexico (Universidad Nacional Autonoma de Mexico-UNAM-), Mexico City, Mexico

**Keywords:** Biomarkers in ovarian cancer, CA125, Survival in epithelial ovarian cancer

## Abstract

**Background:**

Serum levels of CA125 measured before any treatment have been evaluated in epithelial ovarian cancer (EOC) as a predictor of patient survival; however, results in survival index are controversial, as CA125 levels are influenced by several variables. Taking this into consideration, the present study evaluated the association of pretreatment levels of CA125 serum with the clinical stage, histology and differentiation grade of the tumor and the survival rate in a group of patients from an oncology referral center in Mexico, all of them diagnosed with ovarian carcinoma.

This retrospective study consisted of 1009 patients with EOC, diagnosed between 2006 and 2013 at the National Cancerology Institute (Instituto Nacional de Cancerología-INCan), considering only those with CA125 measurements before any chemotherapy or surgical cytoreduction. Patients with three years of medical follow-up having pretreatment CA125 value and simultaneous diagnoses of histological subtype, clinical stage and differentiation grade of the tumor (*n* = 656) were studied in order to determine their survival rate.

**Results:**

The abnormal level (>35 U/mL) of CA125 was observed in 99 % of serous carcinoma cases rated I to IV in the FIGO stages. Abnormal CA125 proportions were 89 % in endometrioid subtype and 69 % in mucinous tumors, with the highest absolute value of CA125 observed in serous carcinoma surpassing any other histological subtype. Clinical stages III and IV displayed increased CA125 values compared to stages I and II. Undifferentiated carcinomas show the highest level of this indicator compared with those of low and moderate differentiated grade. Survival evaluation by Kaplan-Meier analysis including only high grade serous carcinoma at FIGO stage III (*n* = 57) demonstrated 57.1 % chances of survival in patients with CA125 pretreatment levels higher than 500 U/mL. Survival was 26.7 % in patients with CA125 lower than 500 U/mL and the hazard ratio for CA125 ≤ 500 U/mL was 2.28, 95 % CI 1.08–4.84, *P* = 0.032.

**Conclusions:**

Clinical stage associated with pretreatment absolute values of CA125 should be considered as prognostic factor in EOC patients. Values of CA125 higher than 500 U/mL in high grade serous carcinoma with FIGO stage III resulted in an enhanced survival rate of the patients.

## Background

Description of the CA125 antigen determinant represents an important contribution to clinical management of ovarian cancer patients. This glycoprotein recognized by a murine monoclonal antibody (OC125) is codified by MUC16 gene in the locus 19 p13, and is secreted by coelomic derived epithelia [1–4]. Measurement of CA125 in serum are the preferential marker for epithelial ovarian cancer, with CA125 levels higher than 35 U/mL being considered abnormal and associated with 90 % of ovarian carcinoma; levels of CA125 are useful to evaluate the response to chemotherapy, relapse and progression of the disease [5] and measurement of CA125 levels in the screening of ovarian cancer in general population has been largely evaluated [6, 7].

Serum levels of CA125 measured before any treatment or debulking surgery have been evaluated as a reliable predictor of patient survival in epithelial ovarian cancer cases [8]. However, results of initial CA125 levels in relation to survival index are controversial because several variables need to be considered [9]: for example, the percentage of abnormal in CA125 serum levels values increases with the clinical stage of the tumor, reaching to 98 % in FIGO stage IV [4]. Similarly, changes have been observed depending on the histological type of tumors [10].

The aim of this study was to examine the association of pretreatment levels of serum CA125 with the clinical stage, histology and differentiation grade of the tumor as well as the prognostic impact in a group of patients diagnosed with epithelial ovarian cancer (EOC) from an oncology referral center in Mexico.

## Methods

This retrospective study comprised 1009 patients with EOC, diagnosed between 2006 and 2013 at the National Institute of Cancerology (Instituto Nacional de Cancerología-INCan-008/034/OMI) in Mexico City. The protocol of the study was approved by the Ethical Committees of INCan and the School of Medicine of the National Autonomous University of Mexico (Universidad Nacional Autonoma de Mexico-UNAM-108/2015). Only patients with CA125 measurements before any chemotherapy or surgical cytoreduction were considered. Patients with pretreatment CA125 value and simultaneous diagnosis of histological subtype, clinical stage and differentiation grade of the tumor were analyzed (*n* = 656), with the clinical stage, histological subtype and differentiation grade being established according to FIGO and WHO criteria, respectively [11]. Serum levels of CA125 were determined by chemiluminescent enzyme immunoassay (Immunolite OM-MA, Siemens Healthcare), considering CA125 values higher than 35 U/mL as abnormal. Survival analyses included patients with three years’ follow-up from the date of diagnosis. The treatment of patients followed the INCan protocol, including the use of cisplatin and paclitaxel, with all patients being considered for overall survival evaluation notwithstanding the number of chemotherapy cycles received.

## Results

The contingency table with the proportion of normal and abnormal CA125 was analyzed using Pearson’s chi-squared test contrasting the proportions of the groups by calculating “Z” value. Absolute values of CA125 U/mL were normalized by expressing the values as natural logarithms (LN), normal distribution of data was evaluated by Kolgomorov Smirnov’s test and LN data were analyzed by ANOVA with post-hoc Tukey’s test. The survival rate was analyzed using the Kaplan-Meier method and significance by means of log-rank testing. The hazard ratio for CA125 grouped in two categories, low level - 0–500 U/mL- and high level - > 500 U/mL.-, was calculated in serous carcinoma using multivariate Cox regression, the clinical stage and differentiation grade values were considered as covariates. Hazard ratio for the age of the patients has not been included, however, median age of both groups was similar, 53.4 and 53.8 years, respectively.

Significance was considered when the *P* ≤ 0.05. Analysis was performed using SAS version 9.1 (SAS Institute Inc, Cary, NC).

Patients were referred from the whole country, mainly from the central region of Mexico. The educational level of the patients was elementary in three of four. The median age of the study population was 51, ranging from 18 to 90 years old; 60 % of the patients were aged 41–60 years. The mean body mass index of the patients was 26.8 Kg/m^2^, with 60 % of them undergoing menopause.

The following proportions of histological subtypes were observed in epithelial ovarian cancer patients (*n* = 1009): serous 55 %, endometrioid 19 %, mucinous 8 %, clear cells 8 %, and mixed carcinoma 10 %. The clinical stages of the patients were stage III (42 %), stage IV (36 %) whereas stages I and II represented the remaining 22 %.

Considering the degree of differentiation of tumoral cells within the ovarian carcinoma cases studied, undifferentiated carcinoma (G3) was the most frequently diagnosed (67 %), while the moderate differentiated (G2) and well-differentiated (G1) varieties were observed in 17 % and 16 % of the patients, respectively.

Proportion of abnormal CA125 and malignant serum values is indicated in Table 1: Results were grouped by histological subtype and clinical FIGO stage. Analysis of the contingency table indicated that percentage of abnormal CA125 levels changed in relation to histological subtype and clinical stage.

**Table 1:** Pretreatment CA125 level >35 U/mL in epithelial ovarian cancer. Relationship between histological subtype and FIGO stage

	Histological subtypes					
FIGO Stage	Serous	Endometrioid	Mucinous	Clear Cell	Mixed	Total
I	8/9	30/37	16/22	12/18	9/10	75/96
	89 %	81 %	73 %	67 %	90 %	78 %
	231 U/mL	137 U/mL	113 U/mL	153 U/mL	305 U/mL	
II	3/4	6/8	1/1	1/1	2/2	13/16
	75 %	75 %	100 %	100 %	100 %	81 %
	90 U/mL	281 U/mL	75 U/mL	1042 U/mL	252 U/mL	
III	116/118	35/36*	6/10	11/11*	27/28	195/203
	98 %	97 %	60 %	100 %	96 %	96 %
	1255 U/mL	861 U/mL	195 U/mL	415 U/mL	1182 U/mL	
IV	137/137*	13/13	4/6	7/8	10/10	171/174
	100 %	100 %	67 %	88 %	100 %	98 %
	2005 U/mL	511 U/mL	144 U/mL	376 U/mL	1240 U/mL	
Total	264/268	84/94	27/39***	31/38	48/50	
	99 %	89 %	69 %	82 %	96 %	

Values indicate the proportion and percentage of patients with abnormal CA125 and the means of pretreatment values of CA125 U/mL. Comparison of proportions (“Z”)

*P < 0.05 versus FIGO stage I; ***P < 0.001 vs serous and *P* < 0.01 vs. endometrioid total values

Abnormal levels of CA125 were observed in 99 % of serous carcinoma cases ranked from I to IV in their clinical stage progression. Patients with serous carcinoma at clinical stage I displayed normal CA125 values in 11 % of the cases, while in clinical stage IV, all patients with serous subtype showed abnormal CA125 values.

The overall proportion of abnormal CA125 values (stages I to IV) was 89 % in endometrioid subtype of ovarian carcinoma, while patients with an endometrioid carcinoma at FIGO stage I showed 19 % of normal CA125 levels. Similarly to serous subtype, endometrioid ovarian carcinoma displayed 100 % of abnormal CA125 at clinical stage IV.

Reduced proportion of abnormal levels is evident in mucinous subtype ovarian cancer, where the increment of CA125over 35 U/mL was observed in only 69 % of the tumors. Interestingly, the proportion of abnormal CA125 values did not increase in stage III and IV compared to stage I.

Patients diagnosed with clear cells carcinoma displayed an intermediate value between endometrioid and mucinous subtypes, with 82 % of abnormal CA125 values at stages I to IV, showing a significant increase between stage I (67 %) and stages III and IV considered together (95 %).

Mixed carcinoma cases displayed similar values of CA125 to those observed in serous and endometrioid histological subtype. These tumors always include a serous or endometriod component.

**Fig. 1:**
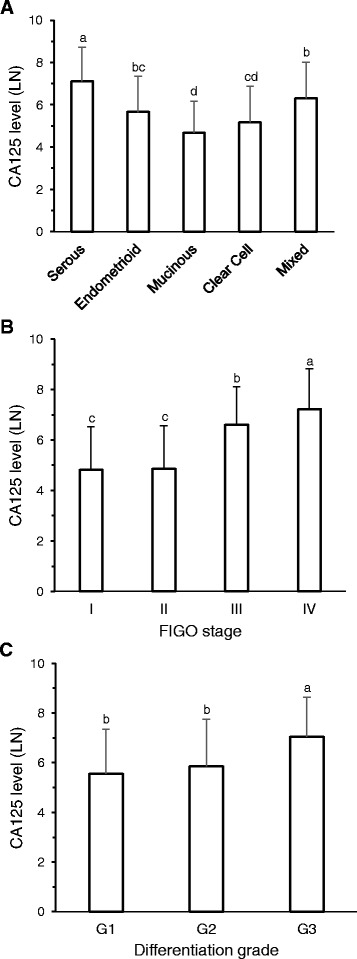
Absolute values of pretreatment CA125 normalized to natural logarithms (LN). **a** Classified by histological subtypes. **b** Grouped according to FIGO stage. **c** Separated by degree of differentiation. Bars represent: Mean ± SD, *n* = as indicated in Table 1. Bars with different superscript indicate *P* < 0.05

The mean serum values of CA125 expressed as U/mL are indicated in Table 1 for each group, classifying the absolute values of CA125 in four levels: 0–35, 35–100, 100–500 and >500 U/mL, CA125 correlated with histological subtype, clinical stage (*p* < 0.001) and differentiation grade (*p* < 0.01). In order to perform an analysis of variance, absolute values were converted to a natural logarithm considering histological subtype, clinical stage and differentiation grade. The highest value of CA125 was observed in serous carcinoma compared to any other histological subtype (Fig. 1a). Clinical stages III and IV displayed increased CA125 values compared to stages I and II (Fig. 1b). Undifferentiated carcinomas show the highest level compared to low and moderate differentiated grades (Fig. 1c).

**Fig. 2:**
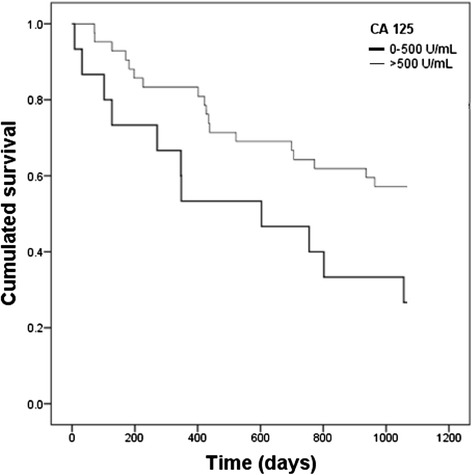
Kaplan-Meier analysis of pretreatment CA125 values separated in low (≤500 U/mL) and high (>500 U/mL) level in patients with high grade serous carcinoma, FIGO stage III. *P* = 0.027 (Log- Rank test)

Survival evaluation was performed by Kaplan-Meier analysis including only high grade serous carcinoma at FIGO stage III (*n* = 57). The comparison was done between low and high pretreatment level of CA125, 0–500 U/mL and more than 500 U/mL, respectively. Results demonstrated a 57.1 % of survival after a three years follow-up of patients with CA125 pretreatment levels higher than 500 U/mL, and although the survival rate was 26.7 % in patients with CA125 lower than 500 U/mL, *P* = 0.027 (Fig. 2). This result was not observed when selection was omitted and the whole population of EOC was included in the analysis (data not shown). The number of patients does not allow the separate analysis of each carcinoma subtype.

The hazard ratio was calculated by means of the Cox regression in patients diagnosed with serous carcinoma (*n* = 85), considering the clinical stage (I to III) and the differentiation grade (G1 to G3) as covariates, resulting in two groups of CA125 values: ≤500 and > 500 U/mL (referent). The result for CA125 ≤ 500 U/mL was HR 2.28, 95 % CI 1.08–4.84, *P* = 0.032.

## Discussion

The patients included in this study constitute a representative sample of the population of the middle region of Mexico and can be considered as roughly similar to the Hispanic population of the United States. All of them came from the same hospital, which is a referral public hospital specialized in oncology cases, and were relatively homogenous in terms of social and economic status. The protocol for diagnosis and treatment followed was the same for all patients. The classification of histological subtype, clinical stage and differentiation grade considered for this study was retrospectively obtained from their clinical records. Measurements of pretreatment serum CA125 were performed at the INCan laboratory or validated there.

The obtained results demonstrated that serous carcinoma displays the highest frequency of abnormal values increasing with the advanced clinical stages (III and IV). Serum absolute values of CA125 increased up to tenfold at FIGO stage IV compared to stage I. A similar observation was obtained in endometrioid carcinoma, with significant low absolute values compared to serous subtype. Mucinous subtype showed abnormal values in 69 % at stage I carcinoma. These results confirm previous findings of several authors [4, 5, 9]. Interestingly, in mucinous carcinoma no increase was observed in relation to clinical stage neither in frequency of abnormal values nor in absolute values of CA125. Observations in mucinous carcinoma have not been explained. It is possible that the secretion of various types of mucins changes the relationship with the MUC16/CA125 expression. Alternatively, the origin of mucinous malignancy explains variations in the behavior of this carcinoma.

Present results show an enhanced survival in patients with high grade serous carcinoma (HGSC), FIGO stage III and pretreatment serum CA125 level higher than 500 U/mL. These results have been observed when the patients were stratified; only HGSC, FIGO III patients were considered for the Kaplan-Meier analysis. The stratified analysis eliminates the influence of histological subtypes, clinical stage and differentiation grade on CA125 levels; this influence was corroborated in the present results. It should be considered that results were obtained classifying over and under 500 U/mL of CA125 serum levels. When the entire cohort of EOC patients was included, Kaplan-Meier analysis showed a poor survival in patients with highest values of CA125, as previously reviewed [5]. Interestingly, a previous study of survival related to WT1 expression in EOC evidenced contrasting changes in the results, considering the entire cohort versus high grade serous subtype in Kaplan-Meier analysis [12]. Similar finding to our results has been previously described in patients stratified by CA125 serum level, the group with CA125 over 2400 U/mL showed a reduction in HR after Cox analysis [13]. Moreover, measuring CA125 in tumor tissue, among late-stage ovarian cancer patients a reduction of hazard ratio (HR = 0.63) has been observed when tumor tissue displayed positive CA125 expression; a positive correlation was found between elevated serum CA125 levels and elevated levels of CA125 tissue expression [14].

The hazard ratio (HR) obtained from Cox regression coincides with Kaplan Meier results, showing an increase of this indicator in patients with CA125 < 500 U/mL. Present results indicate that a low level of pretreatment CA125 in HGSC, FIGO stage III, although abnormal, would have a poor prognostic. The explanation of these results will require further studies; probably, patients with a carcinoma generating high levels of CA125 react differentially to disease or to treatment, resulting in an increased survival rate. Present findings support the theory that EOC is not a single entity, being at least five diseases with different natural histories [15, 16].

## Conclusions

It is important to consider that survival results were obtained from the analysis of a stratified group. Clinical stage associated with pretreatment absolute values of CA125 should be considered a prognostic factor, among others, in EOC patients. Values of CA125 higher than 500 U/mL in high grade serous carcinoma with FIGO stage III resulted in an enhanced survival rate of the patients.

## Abbreviations

EOC, epithelial ovarian cancer; FIGO, International Federation of Gynecology and Obstetrics; HGSC, high grade serous carcinoma; INCan, National Cancerology Institute; WHO, World Health Organization
